# Retroperitoneal liposarcoma in a nonagenarian

**DOI:** 10.4322/acr.2020.224

**Published:** 2020-12-08

**Authors:** King Tung Cheung, Catherine Mitchell, Enoch Wong

**Affiliations:** 1 Monash University, Eastern Health Clinical School, Box Hill, Victoria, Australia; 2 Peter MacCallum Cancer Centre, Department of Pathology, Melbourne, Victoria, Australia

**Keywords:** Sarcoma, Retroperitoneal neoplasms, Colorectal Surgery, Colonic Neoplams, Aged

## Abstract

Retroperitoneal liposarcomas are rare tumors arising from the soft tissue of the retroperitoneum and are of mesenchymal cell origin. They can reach a large size prior to causing symptoms and generally have a poor prognosis. We present the case of a 93-year-old lady presenting with a large retroperitoneal liposarcoma at the site of a previous colonic anastomosis for the adenocarcinoma treatment. It caused minimal symptoms initially, but surgical resection was undertaken when the tumor was found to be growing significantly in size. However, due to the tumor’s location and its invasion into surrounding structures, the resection was not feasible and subsequently abandoned. A retroperitoneal liposarcoma arising from the site of a previous colonic resection has not been previously described. A review of the diagnosis and current management of these lesions is also given.

## INTRODUCTION

Retroperitoneal soft tissue sarcomas (STSs) are rare, accounting for 10-15% of all soft tissue sarcomas.^1^ ^,^ ^2^ Of these STSs, retroperitoneal liposarcomas (RPLs) are the most common subtype.^3^ These lesions often cause minimal or no symptoms and can reach a significant size, growing undetected in the retroperitoneal space before invading or compressing surrounding organs, eventually leading to clinical symptoms. This makes them challenging to diagnose and subsequently treat effectively.

Most RPLs arise de novo but can occur in a pre-existing lipoma. There are no established causative factors but risk factors include ionizing radiation, chemotherapy and some genetic conditions. Trauma, although suspected is not a known risk factor.

We hereby present a case of an RPL occurring at the anastomosis site of a previous left hemicolectomy in a nonagenarian, which has not previously been reported on.

## CASE REPORT

An 88-year-old female underwent a left hemicolectomy for the resection of colonic adenocarcinoma. The histology of the left hemicolectomy showed moderately differentiated colonic adenocarcinoma, with 2 of 6 lymph node involvement i.e. T3N1. She did not receive any adjuvant therapy. Her recovery was uncomplicated, and she was eventually discharged to her general practitioner for ongoing annual surveillance. She had a follow-up colonoscopy, which did not demonstrate any recurrence.

**Figure 1:**
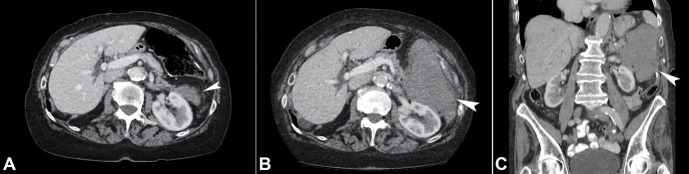
Abdominal CT – **A** – (axial view) showing a 4.7 x 3.0cm soft-tissue density mass anterior to the left kidney, adjacent to the tail of the pancreas and the previous colonic anastomosis at the splenic flexure; **B** – (axial view) showing the rapid expanding tumor, 12.7 x 8.6cm, adherent to the nearby structures; **C** – (coronal view) showing the rapid expanding tumor, 12.7 x 8.6cm, adherent to the nearby structures.

Five years later, at the age of 93, she presented to our service for investigation of a new mass in the left upper quadrant detected on a surveillance abdominal CT. She had only mild non-specific upper abdominal discomfort, and no bowel changes were noted. She also had a history of atrial fibrillation, for which she was on apixaban. No abdominal mass was palpable, and the examination was otherwise unremarkable. The CEA level had been stable and consistently less than 1ug/L (normal non-smoker < 5ug/L). On the abdominal CT, a 4.7 x 3.0cm soft-tissue density mass was evident anterior to the left kidney, adjacent to the tail of the pancreas and the previous colonic anastomosis. (Figure 1A)

A core biopsy of the lesion was performed and showed an atypical spindle cell lesion, thought initially to represent a desmoid or low-grade sarcomatous lesion. It was unable to be characterized further. Given the lack of clinical symptoms, the small size of the lesion, and her advanced age, a decision was made for observation only with interval imaging. Throughout the observation period, the patient remained functionally very well.

However, over a 12-month period, the lesion showed progressive enlargement, up to 12.7 x 8.6cm (Figure 11C).

**Figure 2:**
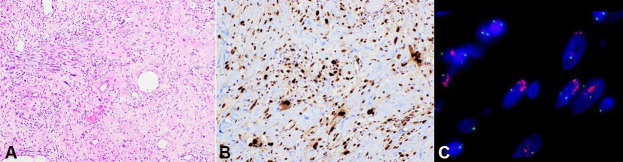
Photomicrographs of the tumor. **A** – showing atypical spindled cells within a collagenous stroma (H&E, x100); **B** – immunohistochemistry shows diffuse reactivity for MDM2 within tumor cell nuclei (x200); **C** – Fluorescence in situ hybridization for MDM2 (12q15) showing amplification of MDM2 (red signals) in comparison with centromere (green signals).

The patient began to complain of the worsening of the abdominal discomfort and fullness. After an extended discussion with the patient and her family, a decision was made to perform a laparotomy in an attempt to excise the lesion. Intraoperatively, the tumor was found to be adherent and inseparable to the nearby structures suggesting local invasion. These included the previous colonic anastomosis, spleen, tail of pancreas and left kidney. The procedure was abandoned due to the potential morbidity of multi-visceral resection to achieve complete surgical excision. Surgical biopsies were performed, which demonstrated a moderately cellular tumor composed of atypical spindle cells within a collagenous stroma (Figure 2A), reactivity for *MDM2* on immunohistochemistry (Figure 2B) and *MDM2* amplification by fluorescence *in situ* hybridization (Figure 2C), consistent with dedifferentiated liposarcoma. The patient was discharged for supportive care and symptom management.

## DISCUSSION

Retroperitoneal liposarcoma is rare and commonly occurs in patients between 40 and 60 years old. It is thought to originate from primitive mesenchymal cells.^4^ Its exact pathogenesis remains unclear but is likely multifactorial. Predisposing factors implicated in other soft tissue sarcomas, including genetic alteration, exposure to radiation and chemical toxins, trauma, and previous surgery, may all play a role.^5^ ^-^ ^7^

Causality between trauma and subsequent development of tumors, especially those of soft tissue origin, has long been suspected. The minimal criteria, as suggested by Warren et al.,^8^ for such association include prior integrity of tumor site, significant tissue disruption, reasonable time frame following the initial injury, and compatibility of tumor type with the reparation and regeneration process of the tissue site.

Retroperitoneal STSs in the form of desmoid tumors developing after surgery have been well described.^9^ ^-^ ^11^ A literature review by Shih et al.^12^ reported retroperitoneal desmoid tumors in at least 12 patients who fit the above minimal criteria. Each of these patients had a variety of intra-abdominal surgeries, from retroperitoneal lymph node dissection to total gastrectomy for GIST. The average time from the previous surgery was 2.3 years, ranging from 11 months to 7 years.

Retroperitoneal STSs occurring in previous surgery sites can often mimic local recurrence of malignancy and pose a challenging diagnostic dilemma. It is important to exclude local recurrence using tumor markers, endoscopy, and other appropriate imaging modalities. On the computed tomography (CT), RPLs generally appear as an encapsulated mass, that contains variable amounts of fatty and soft tissue. CT is most useful in delineating the relationship of adjacent structures, assessing local invasion, and checking for the presence of metastatic disease.^13^

In addition, MRI may demonstrate characteristics of the mass to distinguish between benign and malignant soft-tissue masses. Factors associated with malignant lesions are larger tumor size (> 10cm), thick septa (> 2mm), and less fat content (less than 75%).^14^ Dedifferentiated RPLs often lack macroscopic fat signal intensity. MRI can also assess local tumor extent and surrounding edema, which can then be factored into treatment approaches. It is important to note that the extent of the primary tumor can be underappreciated on imaging studies.

Percutaneous biopsy is controversial as historically, there has been a fear of tumor seeding of the biopsy tract. However, a biopsy is required in cases of diagnostic uncertainty or if neoadjuvant treatment is to be considered. A core biopsy has been shown to be both accurate and safe.^15^ ^,^ ^16^

There are five histologic subtypes of liposarcomas: (i) well-differentiated, (ii) dedifferentiated, (iii) myxoid, (iv) pleomorphic, and (v) liposarcoma, not otherwise specified. More than 93% of all RPLs are of the well-differentiated and dedifferentiated subtypes.^17^ Well-differentiated RPLs are characterized by scattered, often less than 25%, atypical spindle cells among lobules of mature adipocytes showing variation in size. Dedifferentiated RPLs comprise a non-lipogenic, often spindle cell sarcoma, with heterogeneous appearances, which may be associated with a component of well-differentiated liposarcoma. Both well-differentiated and dedifferentiated RPLs are associated with a high rate of local recurrence. Unlike well-differentiated RPLs, which do not tend to metastasize, dedifferentiated RPLs are more aggressive and have a significantly higher risk of metastasis.^18^

Recent advancement in molecular genetics has also shed some light on the genes implicated in liposarcomas. Among these is the *murine double minute 2* (*MDM2*) and *CDK4* genes. *MDM2* encodes a protein that is responsible for the degradation of *p53*, a known tumor suppressor gene, and *CDK4* encodes an oncoprotein that promotes G1/S progression of the cell cycle. Both of these genes are characteristically amplified in well-differentiated and dedifferentiated RPLs.^19^ Overexpression of these proteins are detected by immunohistochemistry or amplification may be detected by fluorescence in-situ hybridization and is useful in distinguishing well-differentiated liposarcomas (WDL) and dedifferentiated liposarcoma (DDL) from other benign and malignant soft tissue tumors.^20^

The treatment of choice for non-metastasized retroperitoneal soft tissue sarcoma is complete surgical excision with negative margins. Complete en bloc excision may require adjacent organ and fat resection, but even then, surgical margins are often narrow.^21^ Unfortunately, even with complete excision, prognosis remains poor, particularly for high-grade RPLs.^22^ Local recurrence is common and can lead to morbidity and mortality.

Preoperative, intraoperative, and postoperative radiotherapy (RT) have all been used to treat RPS, but their role and effectiveness continue to be debated. Preoperative RT tends to be used as the lesion may be accurately targeted and help reduce toxicity to nearby organs, including bowel that may be displaced by the tumor. Furthermore, an unresectable tumor can be converted to a potentially resectable one with the use of RT.^23^

STRASS was a randomized multicenter international trial^24^ comparing preoperative RT followed by surgery to surgery alone. Initial results failed to demonstrate a benefit of preoperative RT for retroperitoneal sarcoma, although the final results are pending. Postoperative RT following complete gross resection has had no study proven value and can be associated with significant toxicities.^25^

Systemic treatments for patients with RPL have mainly occurred in the setting of clinical trials and are typically reserved for those with high-grade tumors. This has been used in both neoadjuvant and adjuvant settings, and doxorubicin, ifosfamide, and anthracycline-based chemotherapy regimens have been used. However, data is limited to small studies, and results have been disparate.

A better understanding of molecular genetics has also led to promising targeted therapy for RPLs. Among others, inhibitors of *MDM2* and *CDK4*, the two most commonly amplified oncogenes, showed some early promising results.^26^ ^,^ ^27^

In the present case, the RPL developed adjacent to the previous colonic anastomosis in the left upper quadrant and was found to be inseparable from the colon and adjacent organs intraoperatively. It is difficult to determine whether the sarcoma developed as a result of the previous surgery or as a primary entity, but its proximity to the previous resection site makes the former far more likely. At the time of the left hemicolectomy, there would have been the dissection of the left colonic and transverse mesentery with a breach into the retroperitoneal region. This may have triggered an abnormal regeneration process to occur, leading to the development of the RPL.

In our patient, stable tumor markers and a recent normal colonoscopy was reassuring and ruled against local recurrence. A diagnosis of a sarcomatous lesion was made after a sample was obtained from a core biopsy of the lesion. Watchful waiting with surveillance scanning was taken initially as the patient was asymptomatic and would be the initial preferred approach in a nonagenerian. However, with the increasing size of the lesion and increase in discomfort, an observational approach was no longer appropriate. The decision to proceed to operative intervention was challenging but after extensive discussion, the patient was agreeable to this, despite her advanced age. At operation, the extent of the RPL was certainly underappreciated on imaging, which was then confirmed to be unresectable due to local invasion.

Decisions such as these in elderly patients are indeed challenging and clearly the risks and benefits of a major operation need to be carefully balanced and explained to the patient. Careful review of the imaging may demonstrate signs of local invasion which may alter the approach and decision making in these complex clinical situations.

## CONCLUSION

To our knowledge, this is the first reported case in which an RPL developed following previous intra-abdominal surgery. RPL may mimic local recurrence of previous malignancy in this setting. Although rare, liposarcoma, and other soft tissue sarcomas, should be kept in mind when investigating potential lesions in the retroperitoneum, particularly in previous surgery settings. Tissue sampling is often required to make the diagnosis. Further studies are needed to consolidate the link between retroperitoneal liposarcoma and previous intra-abdominal surgery. Treatment of RPL is multimodal and should ideally take place in a multidisciplinary center specializing in sarcoma treatment. Complete surgical excision is however, the mainstay of treatment.
